# Sculpted by Love: Attachment Security and the Michelangelo Phenomenon

**DOI:** 10.3390/bs16071237

**Published:** 2026-07-21

**Authors:** Yiwen Gu, Madoka Kumashiro, Ximena B. Arriaga

**Affiliations:** 1 Department of Psychological Sciences, Purdue University, West Lafayette, IN 47906, USA; arriaga@purdue.edu; 2 Department of Psychology and Neuroscience, Goldsmiths, University of London, London SE14 6NW, UK; m.kumashiro@gold.ac.uk

**Keywords:** partner affirmation, personal growth, ideal self, attachment orientation, Michelangelo phenomenon, well-being

## Abstract

The Michelangelo phenomenon describes an interpersonal model of personal growth in which romantic partners affirm each other’s ideal selves, thereby enhancing their relational and personal well-being. The present study examined how attachment security relates to this process. Based on cross-sectional data from *N* = 452 individuals in 258 couples, the results replicated prior work and provided new insights. Greater partner affirmation was associated with higher relationship quality, higher life satisfaction, and lower depression, but was not associated with general anxiety. Movement toward the ideal self mediated the links between partner affirmation and well-being outcomes. Importantly, attachment security (lower attachment anxiety and avoidance) was directly associated with greater partner affirmation and indirectly associated with greater well-being outcomes through partner affirmation and movement toward the ideal self. The effects were particularly robust for lower attachment anxiety, relative to lower attachment avoidance. The findings suggest that attachment security may allow individuals to better rely on close relationships to experience personal growth.

## 1. Introduction

Romantic relationships can be a potent source of personal growth (Feeney & Collins, 2015; Mattingly et al., 2020). Much like the sculptor Michelangelo, who allegedly stated that a sculptor’s role is to help release the ideal figure lying dormant within the marble, a close relationship partner can “sculpt” an individual’s ideal self. The Michelangelo phenomenon encapsulates this process: When individuals feel that a partner affirms their ideal selves, over time they also start to feel that they are moving closer to their ideal selves, which in turn can enhance their relational and personal well-being (Drigotas et al., 1999; Rusbult et al., 2005).

However, not all individuals benefit equally: The Michelangelo phenomenon process may depend on individual differences—for example, in self-regulatory traits (e.g., Kumashiro et al., 2007; Righetti et al., 2010) and other interpersonally oriented traits (Bühler et al., 2020; Gloor et al., 2025). Critically, we posit that individuals may differ in their personal growth based on their attachment orientation, which has been shown to be a powerful predictor of many personal and interpersonal processes, including goal pursuit and social support (e.g., Jakubiak & Feeney, 2016). This research examined couples in a cross-sectional study, both, to replicate and extend previous work on the Michelangelo phenomenon and to advance our understanding of attachment security as a central component of personal growth processes.

### 1.1. An Overview of the Michelangelo Phenomenon

Many individuals, especially in the Western world, are motivated to make progress in their quest to become more like their ideal selves, which reflects aspirations and skills that they intrinsically wish to acquire (Higgins, 1987, 1989; Markus & Nurius, 1986). Goals associated with the ideal self are often pursued individually (e.g., being intrinsically motivated to go to the gym daily to be more fit). However, the literature on goal support also shows the importance of partner support (e.g., their partner may go to the gym with them and/or prepare healthy meals to help the individual get more fit; Fitzsimons & Finkel, 2010).

**Figure 1:**
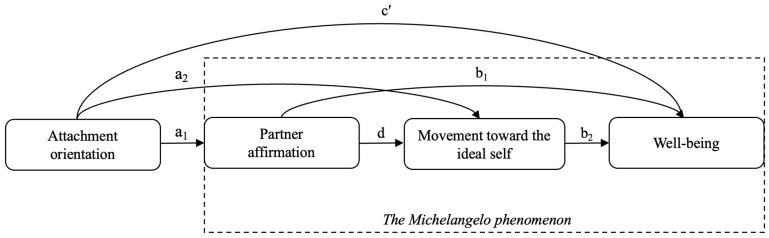
Conceptual Model. *Note*: The Michelangelo phenomenon model appears in the dashed-line box. This research examines whether attachment orientation is associated with relational and personal well-being through the Michelangelo phenomenon processes. The analyses control for each dimension of insecurity (e.g., controlling for avoidance when testing attachment anxiety) and for past ideal self, as explained in the results section. Letters designating specific paths correspond to specific results reported below.

The Michelangelo phenomenon (Drigotas et al., 1999) proposes that individuals can move closer to their ideal selves over time when they perceive that their partners are affirming their ideal selves, as depicted in the boxed area of Figure 1^1^. Specifically, partner affirmation describes the extent to which a partner perceives and behaves toward the individual in ways that match the individual’s ideal self (*perceptual affirmation* and *behavioral affirmation*, respectively), which over time increasingly enables the individual to become their ideal selves (*movement toward the ideal self*). For example, if Mia’s ideal self is to be a confident public speaker, her partner may perceive that she is a good public speaker and behave in ways that encourage her to speak up more, applaud when she speaks up, and comment enthusiastically on the positive aspects of her presentation style. Such interactions over time might make Mia more comfortable with public speaking, thus aligning her current self to closer to her ideal self. In contrast, Mia may not move closer to her ideal self when a close partner draws out other features that are irrelevant to her ideal self (e.g., focusing on Mia’s cooking abilities, which are not important to her) or are antithetical to her ideal self (e.g., seeing her as a poor public speaker and dissuading her from accepting public speaking invitations or criticizing her when she gives a presentation).

Being in a relationship in which individuals perceive that a partner affirms and facilitates movement toward their ideal selves, as opposed to eliciting aspects of the self that are irrelevant or antithetical to their ideal selves, has been shown to enhance both relational well-being and personal well-being (Drigotas et al., 1999; Drigotas, 2002). Prior research has consistently shown that both partner affirmation and movement toward the ideal self are each associated with greater *relational well-being* (Drigotas et al., 1999; Drigotas, 2002; Kumashiro et al., 2007; Rusbult et al., 2009).

However, only three studies to date have examined the Michelangelo phenomenon with respect to *personal well-being*, which seems to be more strongly predicted from partner affirmation than from movement toward the ideal self (Bühler et al., 2019, 2020; Drigotas, 2002). Two of the studies were based on the same sample (Bühler et al., 2019, 2020) and included only one measure assessing satisfaction with life (Diener et al., 1985; German version: Glaesmer et al., 2011). The third study, which relied on a relatively small sample of individuals, reported mixed findings, whereby partner affirmation was associated with life satisfaction, emotional well-being, and loneliness, but movement toward the ideal self was only associated with lower levels of loneliness (Drigotas, 2002).

These mixed findings for the relationship between movement toward the ideal self and personal well-being are puzzling, as it has been well theorized that personal growth striving is a fundamental human motive that should promote personal well-being (e.g., Maslow, 1962; Higgins, 1989; Sheldon & Elliot, 1999). As such, the theory suggests that progressing toward the ideal self should enhance personal well-being, with partner affirmation also providing direct benefits for personal well-being independent of such progress toward an ideal self (e.g., Drigotas, 2002; Kumashiro et al., 2006). Yet, previous findings (Bühler et al., 2019, 2020; Drigotas, 2002) suggest that moving closer to the ideal self may not uniquely contribute to personal well-being.

One aim of the current research was to examine other measures of personal well-being, using a larger dataset. For example, examining depression and general anxiety may be able to capture more specific aspects of well-being related to the ideal self. According to the self-discrepancy theory (Higgins, 1987), which is one of the pillars of the Michelangelo phenomenon (see Drigotas et al., 1999; Kumashiro et al., 2006), discrepancies between the actual self and the *ideal* self as captured here are associated with depression, while discrepancies between the actual self and the *ought* self (i.e., aspects of the self associated with duties and obligations) are associated with general anxiety.

Another aim was to see if associations with personal well-being might be better captured using a different operationalization of movement toward the ideal self than those used in the prior studies. In the three studies that assessed personal well-being (Bühler et al., 2019, 2020; Drigotas, 2002), participants listed their four most desired traits associated with their ideal selves and reported on how much their partner has helped them move closer to these specific traits. However, the ideal self is often viewed as a generalized, global self-concept, rather than as something reflected by a limited set of desired attributes (Higgins, 1989; Markus & Nurius, 1986). We sought to utilize a more abstract measure to assess an overall, global ideal self that includes their desired traits, goals, lifestyle, and a general feeling about how close they are to their ideal selves. Our focus on generalized movement toward an ideal self, rather than on specific aspects of the self, aligns with common approaches to assessing self and relational constructs (e.g., closeness, commitment, self-esteem; Aron et al., 1992; Rosenberg, 1965; Rusbult et al., 1998).

### 1.2. Attachment Orientation and the Michelangelo Phenomenon

We propose that individual differences in attachment orientation should also affect the Michelangelo phenomenon process. Individual differences can shape the experience of the Michelangelo phenomenon. Certain individual differences facilitate partner affirmation and movement toward the ideal self, namely those who are higher on traits associated with self-regulation (e.g., locomotion, an action-oriented trait: Kumashiro et al., 2007; promotion focused: Righetti et al., 2010) or who have more interpersonally adaptive personality characteristics (e.g., agreeableness, emotional stability, extraversion: Bühler et al., 2020; lower narcissism levels: Gloor et al., 2025). With respect to individual difference variables, attachment orientation is one of the most powerful predictors of personal and relational outcomes in the context of close relationships (Mikulincer & Shaver, 2016).

Attachment theory posits that salient interpersonal experiences with close others will shape the internal working models about the self and close others (Bowlby, 1973). These experiences, in turn, govern subsequent expectations, interpretations, emotion regulation tendencies, and behavior patterns in relationship-relevant interactions (Mikulincer & Shaver, 2016). For some individuals, salient past experiences have afforded general trust in others: Individuals who are high in attachment security are comfortable with relational closeness, intimacy, and reliance on partners.

Other individuals have had experiences that do not afford general trust and comfort in close bonds. These differences are captured by assessing adult attachment orientation along two dimensions—attachment anxiety and avoidance—where lower levels on both insecure dimensions indicate greater attachment security (Bartholomew & Horowitz, 1991; Mikulincer & Shaver, 2016). Attachment anxiety is associated with chronic apprehension about being worthy of a partner’s love and persistent attempts to test a partner’s commitment (e.g., seeking constant reassurance, creating drama). Attachment avoidance is associated with resistance to being interdependent with an intimate partner and maintaining distance during emotion-evoking interactions.

How might attachment tendencies affect Michelangelo processes? At the core, these processes involve experiencing and interpreting affirming behaviors and support from a partner within the context of feeling closer to the ideal self and pursuing ideal-relevant goals. Secure attachment has been found to be associated with the belief that a partner is available, responsive, and ready to function as a secure base for goal pursuits (Feeney, 2004) and with being more receptive to a partner’s support (Jakubiak & Feeney, 2016). In addition, greater attachment security has been associated with greater proximity to the ideal self, compared to attachment insecurity (Blalock et al., 2015; Mikulincer, 1995). This suggests that securely attached individuals are more likely than insecure individuals to have affirming close relationship partners who are helping them move closer to their ideal selves—thus supporting the prediction that attachment security is associated with higher levels of Michelangelo processes.

In contrast, attachment insecurity may hinder the extent to which individuals recognize, accept, and benefit from partner affirmation. It is likely that both insecure attachment dimensions may pose barriers to experiencing the positive effects of Michelangelo processes.

Attachment anxiety is strongly associated with a negative model of self (Arriaga et al., 2018; Mikulincer & Shaver, 2016). Anxiously attached individuals may hesitate to pursue independent personal goals because they doubt their own self-worth and fear becoming too independent from the partner (Arriaga et al., 2018), which causes them to underutilize partner support (Collins & Feeney, 2004; Feeney & Thrush, 2010). For example, they may question the sincerity of partner support or interpret support for an independent goal as partner distancing, which then reduces their receptivity to affirmation (Jakubiak & Feeney, 2016). Anxiously attached individuals also may have a less stable and more malleable self-concept (Slotter & Gardner, 2012), which makes it difficult for partners to pin down where to place their support (i.e., which specific features should be affirmed). Therefore, individuals high in attachment anxiety may be less likely than secure individuals to perceive affirmation from their partners because they expect less support and fail to interpret partner support as affirming, and their ever-shifting self-concept may make it difficult to affirm the ideal self.

Attachment avoidance is strongly associated with a mental model that limits dependence and closeness toward a relationship partner (Arriaga et al., 2018; Mikulincer & Shaver, 2016). As attachment avoidance is associated with less self-disclosure (Sun & Jakubiak, 2024), partners may not be able to accurately perceive the kinds of person the individuals aspire to become. Avoidantly attached individuals are also less likely to involve a partner in their important goal pursuits and may prioritize achieving goals independently, reflecting avoidant individuals’ history of learned self-reliance (Feeney & Thrush, 2010). This makes it difficult for partners to provide goal support. Even when partners provide goal support, avoidant individuals may not perceive it as supportive (Collins & Feeney, 2004) and fail to make progress toward their personal goals, compared to secure individuals (Feeney & Thrush, 2010). Therefore, individuals high in attachment avoidance may also be less likely than secure individuals to experience partner affirmation from their partners, because they fail to recognize their partner as a reliable source of support, partners may be unsure of how to support an avoidant partner who keeps their distance, and partners may be eliciting irrelevant aspects unrelated to the ideal self due to lack of disclosure about ideals or inconsistent self-related beliefs (Emery et al., 2018).

### 1.3. Current Research

The primary aim of the current study was to extend research on the Michelangelo phenomenon and attachment theory. This research examined how attachment security (low attachment anxiety, low avoidance) relates to Michelangelo processes (partner affirmation, movement toward ideal self, relational and personal well-being). A second aim was to address mixed findings regarding links between the Michelangelo phenomenon and personal well-being. We used prior assessments of relationship quality and life satisfaction (Drigotas et al., 1999; Drigotas, 2002) but also included additional indicators of well-being, such as depression and general anxiety, which may relate to the ideal self (Higgins, 1987, 1989). A final aim was to introduce a new measure of movement toward the ideal self. Rather than assessing progress toward specific traits, participants reported the extent to which their actual and ideal selves overlap, both currently and prior to their relationship. This approach captures both intrapersonal and relational aspects of the ideal self. The current research was preregistered at: https://osf.io/67g82/overview.

Using a cross-sectional study of romantic couples, the first three hypotheses sought to replicate the past findings on the Michelangelo phenomenon, using different measures of movement toward the ideal self and personal well-being.

**H1.**  *Greater partner affirmation is associated with greater (a) movement toward the ideal self, (b) relational well-being, and (c) personal well-being.*

**H2.**  *Greater movement toward the ideal self is associated with greater (a) relational well-being and (b) personal well-being.*

**H3.**  *Greater movement toward the ideal self mediates the association of greater partner affirmation with greater (a) relational well-being and (b) personal well-being.*

Additional hypotheses provided a novel way to understand the Michelangelo phenomenon as reflecting processes that reinforce attachment security. Because adult attachment security is commonly operationalized as low attachment anxiety and low attachment avoidance, our preregistered predictions were specified separately for each *insecure* attachment dimension. Therefore, negative associations between attachment anxiety or avoidance and partner affirmation indicate that greater attachment security is associated with greater partner affirmation. Although partner affirmation and movement toward the ideal self are theorized to have downstream associations with well-being, we made no *a priori* predictions about the indirect paths from attachment dimensions to well-being through partner affirmation and movement toward the ideal self.

**H4.**  *Greater attachment anxiety is associated with lower partner affirmation.*

**H5.**  *Greater attachment avoidance is associated with lower partner affirmation.*

## 2. Method

### 2.1. Participants

The present study included data from 258 couples who participated during the baseline phase of multi-wave longitudinal projects in a mid-western city in the USA^2^. Individuals and their partners completed a brief online screening survey and were invited to participant in the study if they were 18 or older, lived near each and were in a monogamous relationship of at least 3 months in duration, lived within driving distance of study locations, and spoke English fluently (given that participation meant completing many self-report measures written for English-speaking samples). Couples were drawn from three separate samples (sample sizes: *N* = 182; *N* = 130; *N* = 140). Of these 258 couples, 194 couples had complete data from both partners. The final sample consisted of 452 individual participants (228 female, 221 male, 3 gender non-conforming).

On average, participants were approximately 25.5 years old (*SD* = 9.05). Participants were predominantly heterosexual individuals (94%) who identified as White American (77.4%). The mean relationship duration was 57.10 months (*SD* = 75.39; 6.7% participants reported a duration below 6 months, 7.4% 6–11 months, 60.1% 1–5 years, and 25.8% over 5 years) and almost all participants had regular in-person contact (35.5% reported spending 50 or more hours together per week, 55.9% 20–60 h per week, 9.4% less than 20 h per week). Most participants had at least a high school education and were currently completing their bachelor’s degree (60%) or had their bachelor’s or an advanced degree (40%). Participants from the first sample were younger, less educated, and had been in their relationships for less time than participants in the other two samples, and there were also statistically significant differences across samples in partner affirmation, relationship quality, and life satisfaction. However, these differences were very small in magnitude (η^2^s ≤ 0.03). Importantly, the results of the hypothesis tests did not differ across the three samples^3^.

### 2.2. Procedure

Ethical approval for this research was granted by the Institutional Review Board of Purdue University. Participants were recruited via flyers posted throughout the community and social media advertisements, with payment up to $90 per person for completing the larger study. To be eligible, participants had to be at least 18 years old, in a monogamous relationship for at least three months, and have regular in-person contact with one another (i.e., most participants spend at least 20 hr per week with their partner).

Participants attended a lab session in which they underwent informed consent procedures and then did a series of tasks for a larger study. The first task involved completing questionnaires that included variables analyzed in the current research. The questionnaires took approximately 30 min to complete.

### 2.3. Measures

Self-report measures were used to assess all primary variables. Attachment security was assessed using a measure of adult attachment orientation, and the Michelangelo Phenomenon variables were assessed with measures of partner affirmation, movement toward the ideal self, relational well-being, and three measures of individual well-being (life satisfaction, depression, anxiety). All measures used 7-point Likert scales, except for the measure of movement toward the ideal self, as described below. Higher mean values for each measure represented greater levels of the corresponding variable, with the exception of attachment security as explained below. The full set of the pre-registered measures can be found at https://osf.io/67g82/overview.

*Attachment security*. Participants completed a 12-item scale assessing their attachment orientation toward close relationships in general. The measure included six attachment avoidance items (e.g., “I find it easy to depend on others”; α = 0.91) and six attachment anxiety items (e.g., “I worry that others won’t care about me as much as I care about them”; α = 0.89) that were taken by combining specific items from established scales (ECR-R; Fraley et al., 2000; ECR-RS; Fraley et al., 2006^4^). Lower levels of attachment anxiety and avoidance were used to operationally reflect greater attachment security along these two dimensions.

*Partner affirmation.* Participants completed an 8-item scale assessing the extent to which they perceived their partner as affirming of their ideal selves (e.g., “My partner sees me as the person I ideally would like to be”; α = 0.90; Kumashiro et al., 2007). Perceptual and behavioral affirmation items were combined into one measure tapping into overall partner affirmation, in line with prior research (e.g., Kumashiro et al., 2007).

*Movement toward the ideal self.* A new measure was administered to examine movement toward the ideal self. Similar to the Inclusion of Other in the Self scale (IOS; Aron et al., 1992), participants were shown two diagrams, each with nine pairs of circles varying in the extent of overlap between actual vs. ideal self (1 = no overlap between the actual-self circle and the ideal-self circle to 9 = complete overlap between the two circles). One diagram assessed actual–ideal closeness of self now, whereas the other assessed actual–ideal closeness of self before they became involved with their partner. Movement toward the ideal self was modeled statistically by including current actual–ideal closeness as a predictor, controlling for their actual–ideal closeness before they became involved with their partner^5^.

*Relational well-being.* Participants completed six items assessing their relationship quality (α = 0.83). Five items from the Perceived Relationship Quality Components Inventory (Fletcher et al., 2000) assessed trust (3 items; e.g., “How much do you feel that you can trust your partner?”), satisfaction (“How satisfied do you feel with your relationship?”), and commitment (“How committed to your relationship do you feel?”), and one additional item assessed gratitude (“How grateful are you for your relationship?”).^6^

*Personal well-being.* Personal well-being was conceptualized as reflecting both positive and negative aspects of psychological functioning, by assessing three separate dimensions of life satisfaction, depression, and general anxiety. Two items (from Fisher et al., 2016) assessed life satisfaction (e.g., “I am satisfied with my life”, “In most ways my life is close to ideal”; reliability = 0.83)^7^. Two items (from Löwe et al., 2010) assessed depression (e.g., “I have often been bothered by feeling down, depressed, or hopeless”, “I have often had little interest or pleasure in doing things”; reliability = 0.82). Two items (from Löwe et al., 2010) assessed general anxiety (e.g., “I have often worried about a lot of different things”, “I have often been bothered by feeling tense, nervous, or anxious”; reliability = 0.87).

### 2.4. Data Analysis Strategy

All analyses were conducted in R Studio (Version 4.6.1; Posit Team, 2025) using the Lavaan (Rosseel, 2012) and psych (Revelle, 2024) packages. Correlation and multilevel structural equation modeling were used to test the hypotheses. Because participants were nested within romantic couples, non-independence between partners was accounted for in all regression-based models by specifying couple identification number as a clustering variable.

Serial mediation models were estimated to examine whether the association between the predictor and outcome variables operated through specified mediators in a theoretically driven sequence (VanderWeele, 2016). Models were estimated using maximum likelihood estimation. Indirect effects were estimated with the product of relevant path coefficients, and statistical significance was evaluated based on the corresponding *p*-values (alpha = 0.05). All models that included movement toward the ideal self controlled for past ideal self as a covariate.

## 3. Results

### 3.1. Results Overview

Table 1 provides means, standard deviations, and correlations among key variables. A series of analyses tested specific hypotheses about the relational well-being outcome (relationship quality) and each personal well-being outcome (life satisfaction, depression, and general anxiety).

### 3.2. Hypotheses 1–3: Extension of the Michelangelo Phenomenon

The first three hypotheses aimed to replicate and extend the Michelangelo phenomenon (see the Michelangelo phenomenon dotted-line box in Figure 1), using different measures of movement and personal well-being. Hypotheses 1 and 2 were tested using a series of correlations among partner affirmation, movement toward the ideal self, relationship quality, life satisfaction, depression, and general anxiety (see Table 1). H1 was mostly supported: Individuals who reported greater partner affirmation also reported greater movement toward the ideal self (H1a), relational well-being (H1b), and personal well-being (H1c) for life satisfaction and depression; however, partner affirmation was not associated with general anxiety. Consistent with H2, individuals who reported greater movement toward their ideal selves also reported greater relational well-being (H2a) and personal well-being (H2b: life satisfaction, depression, and general anxiety).

H3 concerned whether movement toward the ideal self mediated the associations between partner affirmation and well-being outcomes. A series of regression models examined the associations between (a) partner affirmation with movement toward the ideal self (path d), and (b) movement toward the ideal self with each outcome (path b_2_), and (c) partner affirmation with each outcome. The models also examined indirect effects through movement toward the ideal self (path d × b_2_), as well as the remaining direct association between partner affirmation and each outcome after accounting for the mediating effect of movement toward the ideal self (path b_1_).

Table 2 presents the results. Consistent with H3a, partner affirmation was positively associated with movement toward the ideal self (path d), *b* = 0.53, *p* < 0.001, which in turn was positively associated with relationship quality (path b_2_), *b* = 0.08, *p* < 0.001. The indirect effect of partner affirmation on relationship quality via movement toward the ideal self was significant (path d × b_2_), *b* = 0.04, *p* < 0.001. The direct effect of partner affirmation on relationship quality remained significant after accounting for the mediator (path b_1_), *b* = 0.36, *p* < 0.001, indicating partial (rather than full) mediation.

Similar results occurred in models testing personal well-being, including life satisfaction (H3b) and depression (H3c), although the direct effect of partner affirmation on depression did not remain significant when accounting for the mediator (path b_1_), *b* = −0.16, *p* = 0.083, indicating full mediation by movement toward the ideal self. For general anxiety (H3d), neither the total effect, *b* = −0.18, *p* = 0.096, nor the direct effect controlling for movement toward the ideal self (path b_1_), *b* = −0.03, *p* = 0.822, was significant. Therefore, the significant indirect effect should be interpreted cautiously given that the total effect between partner affirmation and general anxiety was not significant.

### 3.3. Hypotheses 4 and 5: Attachment Security and Partner Affirmation

H4 and H5 tested the link between attachment security (via each attachment dimension) and partner affirmation (see Figure 1, path a_1_). For each attachment dimension, the association with partner affirmation was tested both with and without controlling for the other dimension (see Table 1, rows 1 and 2; simple correlations without controlling for the other dimension are below the diagonal; partial correlations controlling for the other dimension are above the diagonal).

Consistent with H4, attachment anxiety was negatively correlated with partner affirmation, with and without controlling for attachment avoidance. In tests of H5, the negative association of attachment avoidance was significant when tested as a simple correlation with partner affirmation but became nonsignificant when controlling for attachment anxiety. This provides support for the main prediction that attachment security is positively related to partner affirmation, and the association may be more strongly related to lower attachment anxiety than to lower avoidance.

### 3.4. Attachment Security and the Overall Michelangelo Phenomenon Model

Although no *a priori* hypotheses were made, serial mediation models were conducted to test the direct and indirect associations of attachment security (via each attachment dimension) with Michelangelo phenomenon processes. Specifically, these models examined the associations of (a) each attachment dimension with partner affirmation (path a_1_), (b) partner affirmation with movement toward the ideal self (path d), and (c) movement toward the ideal self with each well-being outcome (path b_2_). The models also tested indirect effects of each attachment dimension via partner affirmation (path a_1_ × b_1_) and movement toward the ideal self (path a_2_ × b_2_) separately initially and then through the full serial pathway (path a_1_ × d × b_2_), as well as the remaining direct association between the attachment dimensions and each outcome after accounting for both mediators (path c′). All serial mediation models controlled for the other attachment dimension as a covariate in predicting well-being outcomes; the models that did not control for it yielded similar results. The results are presented in Table 3 for attachment anxiety and Table 4 for attachment avoidance.

Tests involving attachment anxiety and relationship quality (Table 3) revealed that attachment anxiety was negatively associated with partner affirmation (path a_1_), *b* = −0.09, *p* = 0.001. Partner affirmation was positively associated with movement toward the ideal self (path d), *b* = 0.47, *p* < 0.001. Movement toward the ideal self was positively associated with relationship quality (path b_2_), *b* = 0.06, *p* = 0.001. The indirect effects via partner affirmation (path a_1_ × b_1_), *b* = −0.03, *p* = 0.002, via movement toward the ideal self (path a_2_ × b_2_), *b* = −0.01, *p* = 0.006, and via the full serial pathway (path a_1_ × d × b_2_), *b* = −0.003, *p* = 0.033, all were significant. The direct effect of attachment anxiety on relationship quality was also significant (path c′), *b* = −0.04, *p* = 0.024, indicating partial mediation.

As seen in the additional columns of Table 3, the results were similar for personal well-being, including life satisfaction, depression, and general anxiety, with two exceptions. The indirect effects of attachment anxiety via partner affirmation specifically (path a_1_ × b_1_) on depression, *b* = 0.01, *p* = 0.24, and general anxiety, *b* = −0.00, *p* = 0.864, were not significant.

Tests involving attachment avoidance and relationship quality (Table 4) revealed that greater avoidance was associated with lower partner affirmation (path a_1_), *b* = −0.07, *p* = 0.014. Partner affirmation was positively associated with movement toward the ideal self (path d), *b* = 0.49, *p* < 0.001. Movement toward the ideal self was positively associated with relationship quality (path b_2_), *b* = 0.06, *p* = 0.001. The indirect effects via partner affirmation (path a_1_ × b_1_), *b* = −0.03, *p* = 0.016, and via movement toward the ideal self were significant (path a_2_ × b_2_), *b* = −0.01, *p* = 0.011. However, the indirect effect via the full serial pathway (path a_1_ × d × b_2_), *b* = −0.002, *p* = 0.061, and the direct effect of attachment avoidance on relationship quality (path c′), *b* = −0.03, *p* = 0.102, were not significant, indicating full mediation and minimal downstream effects of avoidance.

For personal well-being, attachment avoidance was negatively associated with partner affirmation (path a_1_), partner affirmation was positively associated with movement toward the ideal self (path d), and in turn, movement toward the ideal self was associated with all three well-being outcomes (path b_2_). However, the indirect effects were less consistent. For life satisfaction, the indirect effects via partner affirmation (path a_1_ × b_1_), *b* = −0.03, *p* = 0.026, via movement toward the ideal self (a_2_ × b_2_), *b* = −0.05, *p* = 0.001, and via the full serial pathway (path a_1_ × d × b_2_), *b* = −0.01, *p* = 0.032, were all significant; however, the direct effect of attachment avoidance (path c′), *b* = 0.01, *p* = 0.857, was not significant. For depression, only the indirect effect via movement toward the ideal self (path a_2_ × b_2_), *b* = 0.06, *p* = 0.001, and via the full serial pathway (path a_1_ × d × b_2_), *b* = 0.01, *p* = 0.032, were significant. For general anxiety, only the indirect effect via movement toward the ideal self was significant (path a_2_ × b_2_), *b* = 0.039, *p* = 0.015. Models without attachment anxiety as a covariate yielded largely similar results, although some total and serial indirect effects became significant, suggesting overlap between the attachment dimensions in their associations with personal well-being outcomes.

In sum, these exploratory results suggest that attachment security is associated with benefits for the Michelangelo phenomenon. While both attachment dimensions contribute to the process, security characterized by low attachment anxiety appears to be a stronger driver for links with personal well-being outcomes than does security characterized by lower attachment avoidance.

## 4. General Discussion

### 4.1. Summary of Results

The current study integrates the attachment literature with the Michelangelo phenomenon by investigating whether and how securely attached individuals experience interpersonal processes that sculpt their ideal selves and foster personal growth and well-being. Several noteworthy findings emerged.

First, the findings mostly supported H1–H3 in replicating and extending prior work on the Michelangelo phenomenon. Greater partner affirmation of the ideal self was associated with greater movement toward that ideal self, which in turn was associated with greater relational and personal well-being. Partner affirmation was indirectly associated with higher levels of all the indices of relational and personal well-being through movement toward the ideal self and directly associated with relational well-being, life satisfaction, and depression, but not robustly with general anxiety.

Second, the findings indicated that attachment security, in particular, lower levels of attachment anxiety, facilitated the Michelangelo phenomenon (see Figure 1) by promoting higher levels of partner affirmation, which had consequences on the rest of the model.

Third, exploratory serial mediation analyses showed that attachment security (lower levels of attachment anxiety and avoidance) was indirectly associated with greater relational well-being and life satisfaction through greater levels of partner affirmation, although the indirect paths (via partner affirmation) did not extend to new personal well-being outcomes of depression and general anxiety. In contrast, attachment security was indirectly associated with all relational and personal well-being outcomes through greater movement toward the ideal self, suggesting the importance of perceiving actual progress toward the ideal self for personal well-being, in addition to having an affirming partner, especially for symptoms of psychological distress.

Notably, the direct and indirect effects were all significant for lower attachment anxiety but not always for lower avoidance once the mediators were included. This suggests that in addition to being affected via Michelangelo phenomenon, attachment anxiety is directly and negatively associated with relational and personal well-being, whereas the negative association of attachment avoidance may be fully absorbed (i.e., mediated) through interpersonal processes.

### 4.2. Broader Implications

The present findings suggest that attachment security, particularly characterized by lower levels of attachment anxiety, may help explain who is more likely to experience affirming support of the ideal self from their partners and subsequent personal growth and well-being. This is consistent with other research that shows positive associations between attachment security and personal growth and well-being processes (Feeney & Collins, 2015).

Whereas attachment anxiety revealed consistent associations, attachment avoidance yielded mixed associations. Thus, the insecurity that disrupts growth and well-being processes may be more strongly driven by attachment anxiety than by avoidance. Might insecure individuals who are highly avoidant but not anxiously attached be somewhat immune to detriments in personal growth? The current results cannot answer to this question, but possible differences in insecure dimensions could be pursued in future research.

What implications do these findings have for insecure individuals? Insofar as the Michelangelo phenomenon bestows well-being via personal growth, it becomes important to understand the conditions that may afford these opportunities for insecure individuals. Recent work identifies specific forms of partner support and specific conditions that may enhance attachment security (Arriaga et al., 2018). The current study implies that insecure individuals may find pathways to greater security via Michelangelo processes—for example, by utilizing strategies to recognize and make use of effective partner support, self-disclosing more or using more effective communication strategies to reveal their goals and aspirations, and/or by framing personal goals as opportunities to overcome less-optimal aspects of oneself. The challenge, of course, is to design effective interventions that can anticipate insecure expectations and interpretations, and can orchestrate effective support (Arriaga & Kumashiro, 2019).

Another implication of the present findings is that well-being outcomes in research on the Michelangelo phenomenon should not be treated as interchangeable. Prior research has shown that partner affirmation and movement toward the ideal self are associated with relational and personal well-being, though effects of partner affirmation are stronger for personal well-being (Drigotas et al., 1999; Drigotas, 2002; Rusbult et al., 2009). However, the weaker evidence for general anxiety in the present study suggests that different well-being indicators may reflect different self-regulatory processes. This pattern is consistent with self-discrepancy theory, which distinguishes actual–ideal discrepancies, involving hopes and aspirations, from actual-ought discrepancies, involving duties and obligations (Higgins, 1987), as well as regulatory focus theory, which distinguishes promotion-focused motivation, driven by growth, aspiration, and the pursuit of gains, from prevention-focused motivation, driven by safety, responsibility, and the avoidance of negative outcomes (Higgins, 1997). Because the Michelangelo phenomenon centers on partner affirmation of the ideal self, it may be more closely tied to promotion-related indicators of well-being, such as greater life satisfaction and lower depressive symptoms rather than prevention-related indicators of well-being, such as threat and general anxiety. Thus, the present findings suggest that well-being outcomes may vary in their theoretical relevance to the Michelangelo phenomenon and should not be assumed to reflect the same underlying process.

Lastly, the present study also has implications for how movement toward the ideal self is conceptualized and measured. A more global measure of movement toward the ideal self, as we used in the current study, may be especially useful because the aspects of the ideal self that feel most salient are likely to vary across time and context. Previous studies have assessed movement toward specific multiple traits or goals associated with the ideal self (e.g., Drigotas et al., 1999), which may not adequately capture the dynamic and shifting nature of the ideal self, as people may hold multiple potential future-oriented self-representations (Markus & Nurius, 1986). Furthermore, the current study used an overlapping-circle measure to capture the multifaceted and dynamic nature of the ideal self. Prior work supports the validity of overlapping-circle measures as indicators of subjective closeness and identification, including self-other overlap in close relationships and self-group overlap in group identity (Aron et al., 1992; Tropp & Wright, 2001; Gächter et al., 2015). This global measure may be especially useful because individuals may emphasize different aspects of the ideal self across time and context, whereas trait- or goal-specific measures often require participants to evaluate progress on pre-specified dimensions. Nevertheless, future research should seek to validate this measure against established indicators of movement toward the ideal self or more static measures of closeness between the actual and ideal self.

### 4.3. Limitations and Directions for Future Research

The current research had several limitations. First, the data were cross-sectional. Although the mediation and serial mediation models were theoretically informed, the present findings cannot establish temporal ordering or causal effects among attachment orientation, partner affirmation, movement toward the ideal self, and well-being (Pirlott & MacKinnon, 2016). Future studies with experimental, longitudinal, dyadic, or daily diary designs may clarify how these processes unfold over time or reflect causal associations.

Second, personal well-being outcomes (life satisfaction, depression, and general anxiety) and relational well-being were assessed with rather brief measures. Although the brief measures were either selected from or modified from validated scales (Fisher et al., 2016; Fletcher et al., 2000; Löwe et al., 2010), they may not capture the full range of personal and relational well-being. Of course, the small effect sizes suggest that processes other than the Michelangelo are likely to explain the negative link between insecure attachment and well-being, such as emotional regulation (Brandão et al., 2020), perceived partner affirmation (Rice et al., 2020), and coping strategies (Altamura et al., 2023). Future studies would benefit from including fuller measures and a broader range of outcomes to determine whether different aspects of the Michelangelo phenomenon are more strongly tied to some forms of well-being than others.

Additionally, a conceptual limitation lies in our operationalization of attachment security. Because standard self-report instruments like the ECR (e.g., Fraley et al., 2000, 2006) measure insecure dimensions of attachment anxiety and avoidance, security in this study is inferred from low levels of attachment anxiety and avoidance. Equating the absence of insecurity to a robust secure attachment style may oversimplify the construct, and future research on adult attachment orientation would benefit from development of validated measures that directly assess attachment security.

Finally, the generalizability of the present findings may be limited. Participants were predominantly White, heterosexual, young college students from the Midwestern United States. Cultural context may shape several components of the present model. Attachment insecurities may relate to support processes differently across cultures, as collectivistic orientations can weaken the negative association between attachment avoidance and perceived support (Frías et al., 2014). Culture may also moderate links among autonomy support, movement toward the ideal self, and well-being: the effect of ideal–actual self-discrepancies on lower relational well-being is stronger in the United States and Russia than in China. Finally, the content of the ideal self may itself be culturally different: promotion-focused self-concepts that are related to growth and aspiration are more often associated with independent or Western contexts and prevention-focused self-concepts that are related to duties and obligations are more often associated with collectivistic or East Asian contexts (Lee et al., 2000; Aaker & Lee, 2001). Thus, future research should examine the Michelangelo phenomenon in more diverse cultural contexts.

## 5. Conclusions

Overall, the present study extends our understanding of how close relationships may contribute to personal growth and relational and personal well-being, and how individual differences in central traits may affect the process. Consistent with past research on the Michelangelo phenomenon (Bühler et al., 2020; Drigotas et al., 1999; Drigotas, 2002; Rusbult et al., 2009), the present study found that partner affirmation and movement toward the ideal self were both positively associated with relational and personal well-being. Importantly, the current research also contributes to the growing literature on the effects of various individual difference traits on the Michelangelo phenomenon (e.g., Gloor et al., 2025; Kumashiro et al., 2007) and to adult attachment theory (e.g., Mikulincer & Shaver, 2016) by suggesting that people who are high in attachment security, especially those low in attachment anxiety, are more likely to experience the Michelangelo phenomenon through perceiving more partner affirmation and movement toward the ideal self. The current findings may thus contribute to understanding why attachment security is associated with higher levels of personal growth and well-being (Blalock et al., 2015; Mikulincer, 1995) and may provide insights for future research on how to develop interventions that buffers the effect of attachment insecurity.

## Figures and Tables

**Table 1:** Means, Standard Deviations, and (Partial) Correlations with Confidence Intervals.

Variable	*M*	*SD*	1	2	3	4	5	6	7	8
1. Attachment anxiety	2.99	1.41			−0.12 ** [−0.21, −0.03]	−0.21 *** [−0.30, −0.12]	−0.17 *** [−0.26, −0.08]	−0.36 *** [−0.44, −0.28]	0.43 *** [0.35, 0.50]	0.34 *** [0.26, 0.42]
2. Attachment avoidance	3.51	1.34	0.26 *** [0.17, 0.34]		−0.08 (*ns*) [−0.17, 0.01]	−0.15 ** [−0.24, −0.06]	−0.12 * [−0.21, −0.03]	−0.06 (*ns*) [−0.15, 0.03]	0.04 (*ns*) [−0.05, 0.14]	−0.05 (*ns*) [−0.14, 0.04]
3. Partner affirmation	5.97	0.82	−0.15 ** [−0.24, −0.06]	−0.11 * [−0.20, −0.02]						
4. Movement toward the ideal self	5.99/4.17	1.43/1.77	−0.26 *** [−0.34, −0.17]	−0.21 *** [−0.30, −0.12]	0.35 *** [0.27, 0.43]					
5. Relationship quality	6.48	0.61	−0.21 *** [−0.30, −0.12]	−0.17 *** [−0.26, −0.08]	0.55 *** [0.48, 0.61]	0.34 *** [0.26, 0.42]				
6. Life satisfaction	5.00	1.34	−0.39 *** [−0.46, −0.30]	−0.15 *** [−0.24, −0.06]	0.34 *** [0.26, 0.42]	0.41 *** [0.33, 0.49]	0.34 *** [0.26, 0.42]			
7. Depression	2.96	1.63	0.45 *** [0.37, 0.52]	0.15 *** [0.06, 0.24]	−0.20 *** [−0.28, −0.11]	−0.38 *** [−0.45, −0.29]	−0.32 *** [−0.40, −0.23]	−0.50 *** [−0.56, −0.42]		
8. General anxiety	4.20	1.83	0.34 *** [0.26, 0.42]	0.04 (*ns*) [−0.05, 0.13]	−0.08 (*ns*) [−0.17, 0.02]	−0.21 *** [−0.30, −0.12]	−0.15 ** [−0.24, −0.06]	−0.36 *** [−0.44, −0.28]	0.65 *** [0.60, 0.70]	

*Note. M* = mean value, *SD* = standard deviation value; values in brackets represent 95% confidence intervals. For movement toward the ideal self, *M* and *SD* values indicate values for closeness to current ideal self/past ideal self. Associations below the table diagonal are zero-order Pearson correlations except for associations involving movement toward the ideal self, which were computed as partial correlations of current closeness to ideal self controlling for past closeness to ideal self. Associations above the table diagonal for attachment anxiety/attachment avoidance were computed as partial correlations controlling for the other attachment dimension. * *p* < 0.05, ** *p* < 0.01, *** *p* < 0.001, (*ns*) not significant.

**Table 2:** Mediation of Partner Affirmation on Well-Being via Movement Toward the Ideal Self.

Path	Relationship Quality	Life Satisfaction	Depression	General Anxiety
	*b*	*b*	*b*	*b*
Affirmation → Movement (d)	0.53 *** [0.38, 0.68]	0.53 *** [0.38, 0.68]	0.53 *** [0.38, 0.68]	0.53 *** [0.38, 0.68]
Movement → Outcome (b_2_)	0.08 *** [0.04, 0.12]	0.33 *** [0.24, 0.41]	−0.43 *** [−0.54, −0.32]	−0.28 *** [−0.42, −0.15]
Indirect (d × b_2_)	0.04 *** [0.02, 0.07]	0.17 *** [0.11, 0.24]	−0.23 *** [−0.32, −0.14]	−0.15 *** [−0.23, −0.07]
Direct (b_1_)	0.36 *** [0.30, 0.42]	0.40 *** [0.26, 0.54]	−0.16 (*ns*) [−0.34, 0.02]	−0.03 (*ns*) [−0.24, 0.19]
Total	0.40 *** [0.35, 0.46]	0.57 *** [0.43, 0.71]	−0.39 *** [−0.57, −0.21]	−0.18 (*ns*) [−0.38, 0.03]

*Note*. Path letters in parentheses indicate paths from Figure 1. Table values are unstandardized coefficients (*b*) with 95% confidence intervals in brackets. All models controlled for past ideal self. *** *p* < 0.001, (*ns*) not significant.

**Table 3:** Serial Mediation of Attachment Anxiety on Well-Being via Partner Affirmation and Movement Toward the Ideal Self.

	Attachment Anxiety:			
Path	Relationship Quality	Life Satisfaction	Depression	General Anxiety
	*b*	*b*	*b*	*b*
Attachment anxiety → Partner affirmation (a_1_)	−0.09 ** [−0.14, −0.03]	−0.09 ** [−0.14, −0.03]	−0.09 ** [−0.14, −0.03]	−0.09 ** [−0.14, −0.03]
Partner affirmation → Movement (d)	0.47 *** [0.32, 0.62]	0.47 *** [0.32, 0.62]	0.47 *** [0.32, 0.62]	0.47 *** [0.32, 0.62]
Movement → Outcome (b_2_)	0.06 ** [0.03, 0.10]	0.26 *** [0.18, 0.35]	−0.33 *** [−0.43, −0.22]	−0.20 ** [−0.33, −0.07]
Indirect via partner affirmation (a_1_ × b_1_)	−0.03 ** [−0.05, −0.01]	−0.03 ** [−0.05, −0.01]	0.01 (*ns*) [−0.01, 0.03]	−0.00 (*ns*) [−0.02, 0.02]
Indirect via movement (a_2_ × b_2_)	−0.01 ** [−0.02, −0.00]	−0.06 *** [−0.09, −0.03]	0.07 *** [0.04, 0.11]	0.04 * [0.01, 0.08]
Serial indirect (a_1_ × d × b_2_)	−0.003 * [−0.01, −0.00]	−0.01 * [−0.02, −0.00]	0.01 * [0.00, 0.02]	0.01 * [0.00, 0.02]
Direct (c′)	−0.04 * [−0.07, −0.01]	−0.26 *** [−0.34, −0.19]	0.43 *** [0.33, 0.52]	0.42 *** [0.30, 0.54]
Total	−0.09 *** [−0.13, −0.05]	−0.36 *** [−0.45, −0.28]	0.52 *** [0.42, 0.62]	0.47 *** [0.35, 0.59]

*Note*. Path letters in parentheses indicate paths from Figure 1. Table values are unstandardized coefficients (*b*) with 95% confidence intervals in brackets. All models controlled for past ideal self and attachment avoidance. * *p* < 0.05, ** *p* < 0.01, *** *p* < 0.001, (*ns*) not significant.

**Table 4:** Serial Mediation of Attachment Avoidance on Well-Being via Partner Affirmation and Movement Toward the Ideal Self.

	Attachment Avoidance:			
Path	Relationship Quality	Life Satisfaction	Depression	General Anxiety
	*b*	*b*	*b*	*b*
Attachment avoidance → Partner affirmation (a_1_)	−0.07 * [−0.13, −0.01]	−0.07 * [−0.13, −0.01]	−0.07 * [−0.13, −0.01]	−0.07 * [−0.13, −0.01]
Partner affirmation → Movement (d)	0.49 *** [0.34, 0.65]	0.49 *** [0.34, 0.65]	0.49 *** [0.34, 0.65]	0.49 *** [0.34, 0.65]
Movement → Outcome (b_2_)	0.06 ** [0.03, 0.10]	0.26 *** [0.18, 0.35]	−0.33 *** [−0.43, −0.22]	−0.20 ** [−0.33, −0.07]
Indirect via partner affirmation (a_1_ × b_1_)	−0.03 * [−0.05, −0.01]	−0.03 * [−0.05, −0.00]	0.01 (*ns*) [−0.01, 0.02]	−0.00 (*ns*) [−0.02, 0.01]
Indirect via movement (a_2_ × b_2_)	−0.01 ** [−0.02, −0.00]	−0.05 ** [−0.08, −0.02]	0.06 ** [0.03, 0.10]	0.04 * [0.01, 0.07]
Serial indirect (a_1_ × d × b_2_)	−0.00 (*ns*) [−0.00, −0.00]	−0.01 * [−0.02, −0.00]	0.01 * [0.00, 0.02]	0.01 (*ns*) [−0.00, 0.01]
Direct (c′)	−0.03 (*ns*) [−0.07, 0.01]	0.01 (*ns*) [−0.07, 0.09]	−0.01 (*ns*) [−0.11, 0.09]	−0.10 (*ns*) [−0.23, 0.02]
Total	−0.07 ** [−0.11, −0.03]	−0.08 (*ns*) [−0.17, 0.01]	0.08 (*ns*) [−0.03, 0.18]	−0.06 (*ns*) [−0.18, 0.06]

*Note*. Path letters in parentheses indicate paths from Figure 1. Table values are unstandardized coefficients (*b*) with 95% confidence intervals in brackets. All models controlled for past ideal self and attachment anxiety. * *p* < 0.05, ** *p* < 0.01, *** *p* < 0.001, (*ns*) not significant.

## Data Availability

Data will be uploaded to OSF upon acceptance.

## Notes

1 Initial research on the Michelangelo phenomenon (e.g., Drigotas et al., 1999; Drigotas, 2002) distinguished between perceptual and behavioral affirmation, positing that the former precedes the latter. Subsequent studies, however, have integrated these processes into a single construct of partner affirmation (e.g., Kumashiro et al., 2007).

2 The same datasets were used in a paper by Rice et al. (2020), although the sample size differed slightly due to reclassification of exclusion criteria and/or missing values for variables that were of specific interest in each study.

3 Gender also revealed some moderations for attachment anxiety’s association with four well-being outcomes. The slopes were more pronounced for men than for women, but the effects for both genders were in the same direction.

4 See Supplementary Material (S1) for scale information and validity.

5 This approach was used as it retains the actual values on the scale more directly than the difference score approach that was originally pre-registered.

6 See Supplementary Material (S2) for scale information and validity. Two items were on a 9-point scale (0–8) in one of the samples. Those scores were recalculated to be on the same 7-point scale as the other items.

7 Each of these personal well-being measures was assessed with two items, warranting use of the Spearman–Brown reliability coefficient rather than Cronbach’s alpha, which yielded comparable results.

## Supplementary Materials

The following supporting information can be downloaded at: https://www.mdpi.com/article/10.3390/bs16071237/s1, Figure S1: Attachment orientation CFA model diagram; Table S1: Fit indices for the attachment CFA; Table S2: Standardized factor loadings for attachment avoidance and anxiety (Fraley et al., 2000, 2006; Lafontaine et al., 2016; Wei et al., 2007); Figure S2: Relationship quality CFA model diagram (Fletcher et al., 2000; Algoe, 2012; Gordon et al., 2012); Table S3. Fit indices for the relationship quality CFA; Table S4. Standardized factor loadings for relationship quality.
