# Supplementary material for: Sculpted by Love: Attachment Security and the Michelangelo Phenomenon
Source: Behav Sci (Basel). 2026 Jul 21;16(7):1237. doi: 10.3390/bs16071237 (PMC13403956; doi:10.3390/bs16071237)
Supplement: Supplementary file 1 [file behavsci-16-01237-s001.zip › behavsci-4345621-supplementary.pdf]

Supplementary Material
Scale information and confirmatory factor analyses for
attachment orientation and relationship quality

This supplement reports scale information and confirmatory factor analyses (CFAs) for the attachment orientation and relationship quality measures. The measures were preregistered prior to analysis (https://osf.io/67g82/overview accessed on 13 May 2026). Model specifications, diagrams, fit indices, standardized factor loadings, and rationales for correlated residuals or method factors are provided below. All models were estimated with N = 452.

Supplement S1. Attachment Security

Scale Information

Attachment security was measured using the full validated short 9-item measure of ECR-S (Fraley et al., 2006), comprising of 6 items for the avoidance dimension and 3 items for the anxiety dimension. Given the shortness of the attachment anxiety dimension compared to attachment avoidance, we included an additional 3 items for attachment anxiety (Anxiety items 10-12) from the fuller ECR-R scale (Fraley et al., 2000) to capture the broader experiences of attachment anxiety. This is in line with various research that has attempted to find the best short form of assessing adult attachment orientation (e.g., Fraley et al., 2006; Lafontain et al., 2016, Wei et al., 2007). As is the practice in assessing attachment security in adult close relationships, attachment security is represented by lower levels of attachment anxiety and avoidance. Scale items can be found in the CFA below.

Attachment Security CFA

Model specification

Attachment security was modeled using two correlated latent factors: attachment avoidance and attachment anxiety. Avoidance was indicated by #1r through #6, with #1 to #4 reverse-scored (adding “r”) so that higher scores reflected greater avoidance. Anxiety was indicated by #7 through #12. Because the avoidance indicators included both reverse-scored positively worded items and directly negatively worded items, an orthogonal positive-wording method factor was added for the four reverse-scored items.

Figure S1. Attachment orientation CFA model diagram

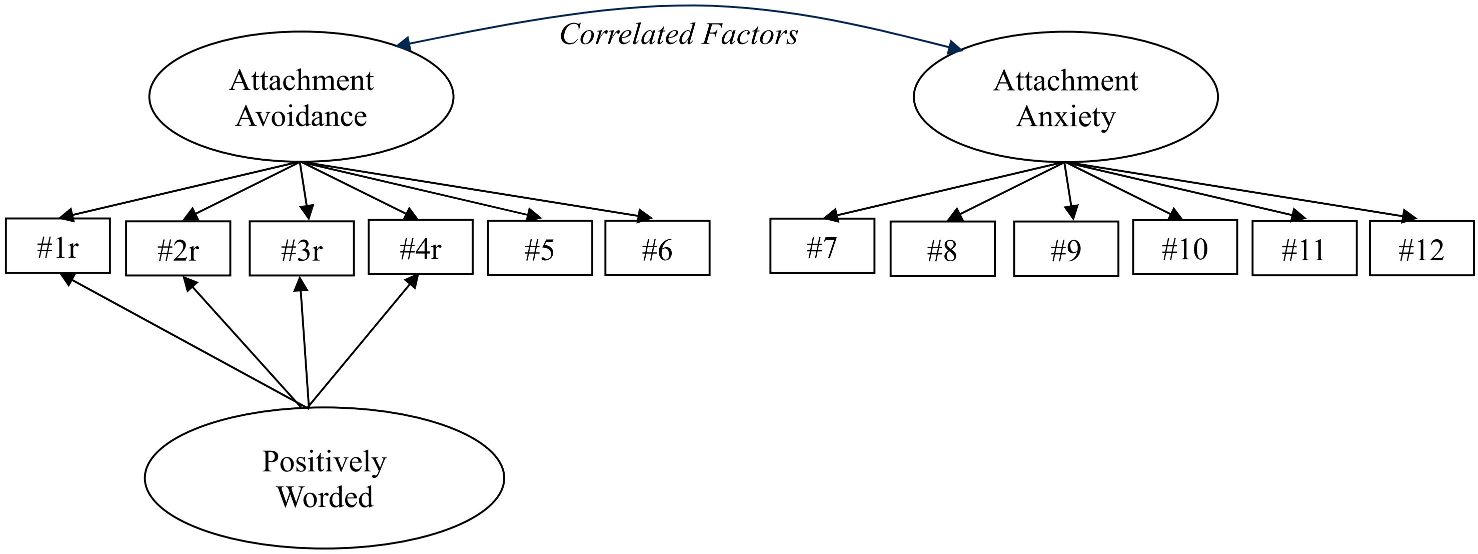

Table S1. Fit indices for the attachment CFA

chi-square	df	p	Robust CFI	Robust TLI	Robust RMSEA	90% CI for RMSEA	SRMR
191.13	49	< .001	.953	.937	.087	[.074, .100]	.053

Note. This table summarizes the final two-factor attachment model with an orthogonal positive-wording method factor. Robust CFI, robust TLI, and robust RMSEA are reported; the chi-square value is the scaled test statistic.

Standardized factor loadings

Table S2. Standardized factor loadings for attachment avoidance and anxiety

Item	Item content	Std. loading
Avoidance: #1r	It helps to turn to others in times of need.	.532
Avoidance: #2r	I usually discuss my problems and concerns with others.	.655
Avoidance: #3r	I talk things over with others.	.607
Avoidance: #4r	I find it easy to depend on others.	.555
Avoidance: #5	I do not feel comfortable opening up to others.	.858
Avoidance: #6	I prefer not to show others how I feel deep down.	.892
Anxiety: #7	I often worry that others do not really care for me.	.840
Anxiety: #8	I am afraid that others may abandon me.	.812
Anxiety: #9	I worry that others will not care about me as much as I care about them.	.872
Anxiety: #10	When I show my feelings toward others, I am afraid they will not feel the same about me.	.863

Anxiety: #11	I find that others do not want to get as close as I would like.	.726
Anxiety: #12	Sometimes others change their feelings about me for no apparent reason.	.621

Note. Avoidance #1-6 and Anxiety #7-9 are from the validated 9 item ECR-S scale (Fraley et al., 2006), while Anxiety #10-12 were taken from the 18-item anxiety dimension of ECR-R (Fraley et al., 2000).

Rationale for the method factor

The positive-wording method factor was included to account for shared wording-related variance among the four reverse-scored avoidance items. These items were positively worded in their original form but reverse-scored so that higher scores reflected greater avoidance. Because these items differ in wording direction from the directly worded avoidance indicators, they may share response-set or wording-related variance that is not part of the substantive avoidance construct.

This approach is consistent with prior work on the ECR-S. Wei et al. (2007) reported that the two-factor anxiety/avoidance structure fit adequately after accounting for method effects related to item wording. Thus, the present model follows the same general logic with the avoidance and anxiety factors represent the substantive attachment dimensions, and the positive-wording factor captures wording-related variance.

Supplement S2. Relationship Quality

Scale Information

The relationship quality measure was conceptualized using a higher-order framework based on the Perceived Relationship Quality Components (PRQC) Inventory (Fletcher et al., 2000). Fletcher et al. demonstrated via CFA that perception of relationship quality is comprised of distinct first order constructs such as trust, commitment, and satisfaction, that load onto a higher-order single factor of perceived relationship quality. Fletcher et al. also explicitly validated a short version of the inventory (comprising 1 item per domain), suggesting that single-item indicators of these facets reliably capture the overarching variance of relationship quality.

This study utilized baseline measures from existing longitudinal datasets on strengthening attachment security, in which we were particularly interested in the trust, commitment, and satisfaction dimensions of PRQC. Thus, we used 5 items from PRQC, maintaining the full 3-item subscale for trust (the central construct of interest), as well as retaining single-item indicators for satisfaction and commitment. In addition, we included a single item on gratitude, which has been shown to be a key driver of relationship well-being and partner responsiveness (e.g., Algoe, 2012; Gordon et al. 2012). The six scale items can be found in the CFA below.

Relationship Quality CFA

Model specification

Relationship quality was modeled as a single latent factor indicated by six items assessing trust, ability to count on the partner, partner dependability, gratitude, satisfaction, and commitment. The six indicators were #1, #2, #3, #4, #5, and #6. Because #1, #2, and #3 were all trust-related items with highly overlapping item content, we used residual correlations among these three indicators. The model was estimated using maximum likelihood with robust standard errors.

Figure S2. Relationship quality CFA model diagram

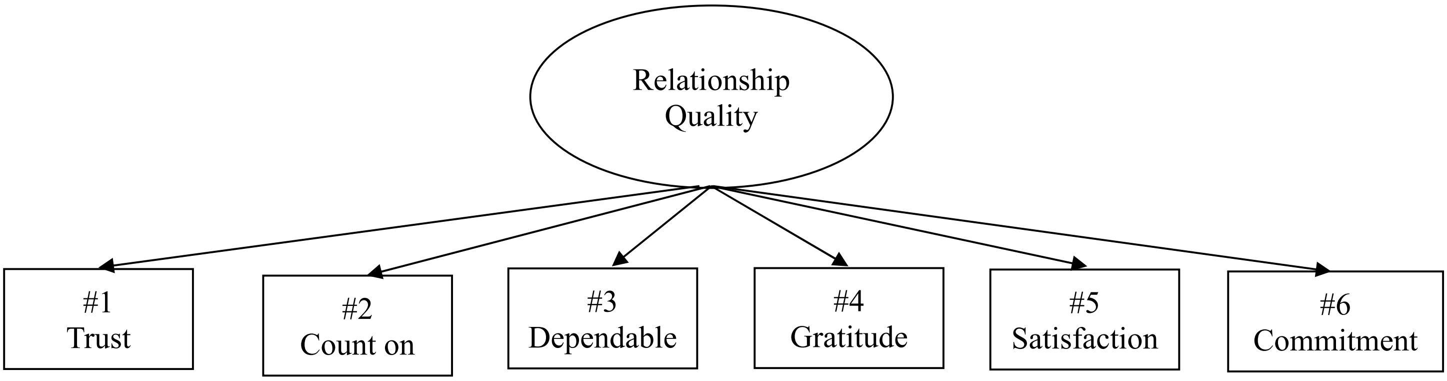

Correlated residuals among trust-related items
#1 ~~ #2
#1 ~~ #3
#2 ~~ #3

Table S3. Fit indices for the relationship quality CFA

chi-square	df	p	Robust CFI	Robust TLI	Robust RMSEA	RMSEA 90% CI for RMSEA	SRMR
15.88	6	.014	.984	.960	.080	[.028, .132]	.032

Note. This table summarizes the one-factor model with correlated residuals among #1, #2, and #3. Robust CFI, robust TLI, and robust RMSEA are reported; the chi-square value is the scaled test statistic.

Standardized factor loadings

Table S4. Standardized factor loadings for relationship quality

Item	Item content	Std. loading
#1	How much do you feel that you can trust your partner?	.569
#2	How much do you feel that you can count on your partner?	.628
#3	How much do you feel that your partner is dependable?	.500
#4	How grateful are you for your relationship?	.758
#5	How satisfied do you feel with your relationship?	.756
#6	How committed to your relationship do you feel?	.643

Note. #1-#3 (trust dimension) and #5-#6 are from the PRQC Inventory (Fletcher et al., 2000), while #4 was created for the project.

Rationale for correlated residuals

We used correlated residuals among #1, #2, and #3 because these three items comprise the trust dimension of the original PRQC and assess trust-related perceptions of the partner: trusting the partner, being able to count on the partner, and perceiving the partner as dependable.

Supplement References

Algoe, S. B. (2012). Find, remind, and bind: The functions of gratitude in everyday relationships. *Social and Personality Psychology Compass*, *6*(6), 455-469.

Fletcher, G. J. O., Simpson, J. A., & Thomas, G. (2000). The measurement of perceived relationship quality components: A confirmatory factor analytic approach. *Personality and Social Psychology Bulletin*, *26*(3), 340–354.

Fraley, R. C., Niedenthal, P. M., Marks, M., Brumbaugh, C., & Vicary, A. (2006). Adult attachment and the perception of emotional expressions: Probing the hyperactivating strategies underlying anxious attachment. *Journal of Personality*, *74*(4), 1163–1190.

Fraley, R. C., Waller, N. G., & Brennan, K. A. (2000). An item-response theory analysis of self-report measures of adult attachment. *Journal of Personality and Social Psychology*, *78*, 350-365.

Gordon, A. M., Impett, E. A., Kogan, A., Oveis, C., & Keltner, D. (2012). To have and to hold: Gratitude promotes relationship maintenance in intimate bonds. *Journal of Personality and Social Psychology*, *103(2)*, 257–274.

Lafontaine, M.-F., Brassard, A., Lussier, Y., Valois, P., Shaver, P. R., & Johnson, S. M. (2016). Selecting the best items for a short-form of the Experiences in Close Relationships questionnaire. *European Journal of Psychological Assessment, 32*(2), 140–154.

Wei, M., Russell, D. W., Mallinckrodt, B., & Vogel, D. L. (2007). The experiences in close relationship scale (ECR)-short form: Reliability, validity, and factor structure. *Journal of Personality Assessment*, *88*(2), 187–204.
